# The Association of Eating Behaviour with Physical Activity and Screen Time among Adolescents in the Klang Valley, Malaysia: A Cross-Sectional Study

**DOI:** 10.3390/healthcare11091260

**Published:** 2023-04-28

**Authors:** N. Z. M. Saat, Siti Aishah Hanawi, Nurul Hasanah Hasmuni Chew, Mahadir Ahmad, Nor M. F. Farah, Masne Kadar, Hanis Mastura Yahya, Nor Malia Abd Warif, Muhammad Khairuddin Md Daud

**Affiliations:** 1 Programme Biomedical Science, Centre of Community Health (ReaCH), Faculty of Health Sciences, Universiti Kebangsaan Malaysia, Jalan Raja Muda Aziz, Kuala Lumpur 50300, Malaysia; 2 SOFTAM, Faculty of Information Science and Technology, Universiti Kebangsaan Malaysia, Bangi 43600, Malaysia; 3 Dietetics Programme Centre of Community Health (ReaCH), Faculty of Health Sciences, Universiti Kebangsaan Malaysia, Jalan Raja Muda Aziz, Kuala Lumpur 50300, Malaysia; 4 Clinical Psychology & Behavioural Health Program, Center for Community Health Studies (ReaCH), Faculty of Health Sciences, Universiti Kebangsaan Malaysia, Jalan Raja Muda Aziz, Kuala Lumpur 50300, Malaysia; 5 Programme of Occupational Theraphy, Centre for Community Health Studies (ReaCH), Faculty of Health Sciences, Universiti Kebangsaan Malaysia, Kuala Lumpur 50300, Malaysia; 6 Occupational Therapy Programme, Centre for Rehabilitation and Special Needs (iCaRehab), Faculty of Health Sciences, Universiti Kebangsaan Malaysia, Jalan Raja Muda Abdul Aziz, Kuala Lumpur 50300, Malaysia; 7 Dietetics Programme and Centre for Healthy Ageing and Wellness (H-CARE), Faculty of Health Sciences, Universiti Kebangsaan Malaysia, Jalan Raja Muda Abdul Aziz, Kuala Lumpur 50300, Malaysia; 8 Programme of Biomedical Science, Center for Toxicology & Health Risk Studies, Faculty of Health Sciences, Universiti Kebangsaan Malaysia, Jalan Raja Muda Abdul Aziz, Kuala Lumpur 50300, Malaysia

**Keywords:** eating behaviour, physical activity, screen time, adolescents

## Abstract

Over the last few decades, the time adolescents spend using electronic devices has increased significantly. The aim of this study was to evaluate the impact of screen time and physical activity on eating behaviour in adolescents. This study used the Physical Activity Questionnaire for Adolescents (PAQ-A) Malay version and the Dutch Eating Behaviour Questionnaire (DEBQ) Malay version methods with secondary students around the Klang Valley. The sampling technique used was purposive sampling. With parents’ consent, an online survey was conducted among adolescent school children aged between 13 and 17 years during the COVID-19 pandemic in the Klang Valley, Malaysia. There were 372 respondents participating in this study. The results showed that 99.4% of them had more than 4 h screen time a day, and that 38.5% have more than three devices at home. Moreover, there was a significant mean difference in screen time for console games without a handheld device between male and female adolescents (*p* < 0.05). There was also a significant mean difference in the emotional, restricted and external eating behaviour scores between male and female adolescents (*p* < 0.001). There was a significant relationship between physical activity and the time duration spent on a television, telephone and laptop during weekends (*p* < 0.05). There was a significant relationship between eating behaviour and time spent watching television and using laptops during weekends (*p* < 0.05). Based on the moderation model, gender as a moderator variable indicated that there was a significant relationship between screen time and interaction screen time and gender with emotional eating (*p* < 0.001). Female adolescents had a stronger relationship between screen time and emotional eating compared to male adolescents (*p* < 0.05). Meanwhile, for physical activity level as a moderator variable, the results showed that there was a significant interaction between screen time and physical activity for emotional eating behaviour (*p* < 0.05). In conclusion, this study indicates that screen time was higher among female adolescents and significantly related to emotional eating behaviour. Therefore, the educational sector should emphasise the motivation of adolescents to engage in physical activity, reduce their screen time and eat healthy foods such as fruits and vegetables.

## 1. Introduction

The COVID-19 pandemic has contributed to lifestyle changes among adults and adolescents. Nowadays, adolescents prefer to communicate online with their peers. This may be because during the COVID-19 pandemic, most people met online, especially for online learning and discussion, to prevent the disease from spreading. Thus, the use of gadgets in their daily routines has become common for adolescents, and having a gadget such as a smartphone has become the norm [1,2].

Screen time can be defined as time spent at a screen either watching television, playing video games, or using a smartphone for surfing the internet and other activities. A maximum of 2 h screen time per day for adolescents was recommended based on a previous study. However, most adolescents spent more than 2 h per day looking at a screen, according to studies in Brazil 79.5% and Canada 47.52% [3,4]. Another study in India reported that 68% adolescents had more than 2 h screen time daily, involving screen-based media such as television, mobile phones, tablets and laptops [5,6]. Previously, a local study conducted in the central region of Malaysia indicated that on average, 3.67 h was the screen time among adolescents, which is more that the average 2 h that was recommended by the American Academy of Pediatrics (AAP) [7,8]. Another study conducted in Malaysia discovered that adolescents spent 3.1 h per day on average viewing screens [9]. Recent thorough research with children and adolescents has additionally indicated that during the COVID-19 pandemic, the use of portable devices and personal computers grew by 52%, with this increase being most prominent in youngsters between the ages of 12 and 18 [10].

The high percentage of adolescents exposed to screen time is of concern because screen time can increase the risk of obesity, cardiovascular disease and being physically inactive, whilst producing changes in dietary habits. A meta-analysis has illustrated that a longer time spent at a screen per day is associated with a 1.67 times increased risk of childhood obesity and being overweight, compared to children who had less than 2 h per day screen time [11]. A longitudinal study among adolescents has also revealed that as screen time increased, BMI also increased, among adolescents aged between 14 to 18 years [12]. Another study in Greece reported that increasing screen time significantly increased the likelihood of unhealthy dietary habits such as consuming fast food, eating sweets regularly, reduced fruit and vegetable intake, skipping breakfast and other dietary factors [13] in the United Kingdom [14]. Another previous study indicated that there was a significant mean difference in consumption of food groups—sweet drinks, sweets, fruit and fast foods—between normal and overweight groups of adolescents in Italy [15].

Adolescent eating behaviours tend to reflect excessive screen time and low physical activity. Prior studies have discovered there is a positive relationship between emotional eating with BMI in cross-sectional and longitudinal studies. A study among adolescents in Canada reported that there was a positive relationship between the score for emotional eating and total screen time, whilst a negative relationship was found between restrained eating and total screen time [16]. Additionally, high emotional eating behaviour was related to significantly higher intake of fast food, high-fat snacks, processed meat and desserts compared to low emotional eating behaviour, in a study conducted among adolescents [17]. In another study in the United States, emotional eating was related to frequent intake of high-salt, high-energy density sweets and soda [18]. According to a study conducted in Finland, eating processed food, chocolates, and sweets among boys is significantly associated with eating behaviour related to stress. Girls tended to consume chocolate, candies, and sugar-free soft drinks during stressful times. [19]. 

Furthermore, referring to previous studies, eating behaviour, screen time and physical activity appear to be interrelated, affecting adolescents. However, there is a lack of research aiming to understand this relationship alongside the COVID-19 pandemic in Malaysia. Hence, the aim of this study was firstly to evaluate the differences in screen time with different appliances, eating behaviours and physical activity levels between genders during the weekend and on weekdays. The second aim was to determine the relationship between screen time and eating behaviour and physical activity, and to determine the moderating effect of gender and physical activity on the relationship between the behaviour eating score and screen time. 

## 2. Materials and Methods

### 2.1. Study Design

This study is a cross-sectional study using the convenience sampling method. In this study, three questionnaires were used and developed using Google Forms. The questionnaire was self-administrated. The location of the study was the state of Selangor, Malaysia. The research was conducted from March 2021 until October 2022.

### 2.2. The Inclusion and Exclusion Criteria

The inclusion criteria were adolescents aged 13 to 17 years old who have a gadget or digital devices such as a smartphone, laptop, desktop, etc. Furthermore, the respondents were given the consent of their parents or guardians to participate in this study, and the form was signed digitally. The exclusion criteria were adolescents with a physical disability, who had been diagnosed with a food disorder such as anorexia nervosa, bulimia nervosa, etc., or who were on medication or diagnosed with a mental illness. 

### 2.3. Instruments

The instruments that were used in this study addressed socio-demographic questions, DEBQ, screen time and PAQ-A. The socio-demographic questions consisted of gender, age, race, location and household income. The screen time questions sought information on the number of devices in the home, the type of devices and the duration of use for every digital device during weekends and weekdays. Total screen time was calculated based on total time using devices.

The Dutch Eating Behaviour Questionnaire (DEBQ) has been translated by a previous researcher into the Malay language. DEBQ consists of a 33-item questionnaire divided into three eating behaviour disorder sections: emotional eating [13 questions], external eating [10 questions] and restrained eating [10 questions]. The response was via a 5-point Likert scale [1 ‘never’ up to 5 ‘very often’]. The internal consistency was more than 0.914 for emotional eating, 0.786 for external eating and 0.856 for restrained eating [20]. The scoring was the summation for every section, and the average was calculated to determine the level of eating behaviour. 

The Physical Activity Questionnaire (PAQ-A) was also in the Malay language. PAQ A consisted of 9 items to measure the physical activity among adolescents. The items in the questionnaire concerned the type of activity engaged in during leisure time, such as skipping, running, cycling and others. There was also a question about physical activity classes in the past 7 days. The other question was on physical activity during respondents’ spare time, recess time in school, during weekends and within their daily routine [21,22]. The scoring of PAQ-A was based on mean scoring for overall 8 items. A mean less than 1 indicated low physical activity, and a mean of 5 and above indicated the participant was physically active [23].

#### 2.3.1. Sample Size

The sample size was calculated using the formulae *n* = z_α/2_ *p* (1 − *p*)/D^2^, *n* = (1.96)^2^ × (0.51) × (1 − 0.51)/0.05^2^, *n* = 384 [24]. The prevalence *p* of sedentary lifestyle among adolescents in Malaysia was 51.9%, based on previous studies [25]. 

#### 2.3.2. Ethical Issues

This study was approved by the Research Ethics Committee of Universiti Kebangsaan, Malaysia (JEP-2021-503).

### 2.4. Statistical Analysis

In this study, data were extracted from the Google Form as a spreadsheet after the data collection was complete. A descriptive analysis was performed to describe the profile of the respondent. Next, to compare the total score of the eating behaviour between gender, an independent t test was performed. Hierarchical regression was used in order to evaluate the relationships between the total score for DEBQ during weekends and weekdays, and the total PAQ-A score during weekends and weekdays with the screen time duration using different devices. Lastly, for the moderating variables of gender and physical activity level, the moderating analysis Hayes process was used to determine the relationship between screen time and eating behaviour. Data were analysed using SPSS version 24 [26].

## 3. Results

### 3.1. Characteristics of the Respondents

There were 372 respondents participating in this study. Table 1 revealed the respondents were mostly female adolescents (62.2%). The age of participants was mostly between 16 to 17 years (75%). Most commonly, the household income was RM1001 to RM3000. Most of the subjects were Malay (85%), followed by Indian (10.2%), Chinese (4%) and others (0.6%). More than half of the adolescents have less than three devices at home (62.9%), and almost all of them accumulated more than 2 h screen time a day (99.5%). 

### 3.2. The Effect of Screen Time during Weekends and Weekdays between Gender

Table 2 shows the findings on the average differences in screen time by device type. Between genders, there was a significant mean difference in non-handheld console games played on weekdays (*p* < 0.001) and weekends (*p* < 0.05). However, there was no discernible mean difference found in the amount of screen time for other devices (*p* > 0.05) between male and female adolescents using an independent t test. Table 3 shows that there were significant mean differences in the scores for the eating behaviour and emotional (*p* < 0.001), restricted eating (*p* < 0.001), and external eating (*p* < 0.001) domains between genders. Additionally, the mean difference in physical activity levels between genders was significant (*p* <0.001).

### 3.3. Relationship between Screen Time and Eating Behaviour

Table 4 suggests that eating habit scores were associated with the hours spent on a desktop device (β = 0.080 (0.037), *p* = 0.034), and also that they had an inverse association with time spent using a game console (β = −0.90 (0.035), *p* = 0.011) during weekdays. Moreover, during weekends, eating habit scores were associated with duration watching television (β = 0.043 (0.20), *p* = 0.31) and duration using laptop (β = 0.058 (0.026), *p* = 0.026). Besides that, this study also found that physical activity levels during weekdays were associated with spending time using a tablet (β = 0.112 (0.04), *p* = 0.006). Meanwhile, physical activity levels during the weekends were significantly associated with decreased time spent on telephones (β = −0.023 (0.009), *p* = 0.010), increased time spent watching television (β = 0.031 (0.014), *p* = 0.025), and time spent using a laptop (β = 0.044 (0.019), *p* = 0.024).

### 3.4. Prediction of Eating Behaviour by Screen Time and Gender with Physical Activity Level as a Moderating Variable

**Figure 1:**
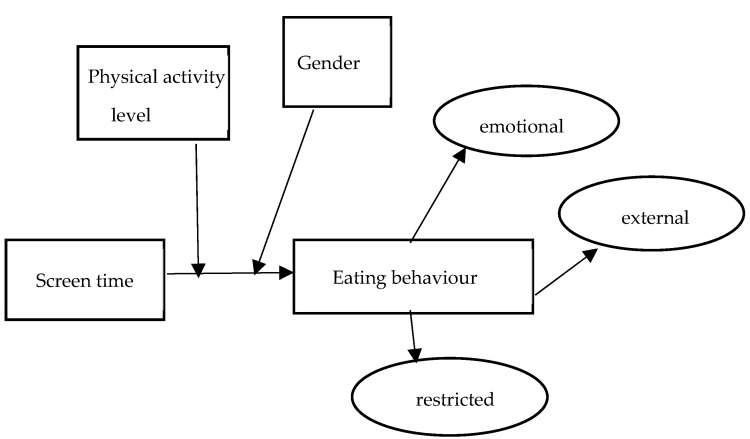
The proposed moderation model demonstrating the conditional effect of screen time on eating behaviour among adolescents on the basis of gender.

The proposed moderation model is described in Figure 1. The moderation model used in Table 5 for significant variance among adolescents reported emotional eating R^2^ = 0.1416, *p* < 0.001. A combined effect of this magnitude (f^2^ = 0.016) was considered a small- effect size [27]. The interaction term (XW) accounted for a significant 1.4% of the variance in the emotional eating variable (F = 5.0325, *p* < 0.05). There was a significant moderation effect of gender, suggesting that female adolescents had a stronger positive relationship between screen time and emotional eating (B_female_ = 0.3784, *p* < 0.01) compared to male adolescents (B_male_ = 0.0496, *p* > 0.05). Meanwhile, for external eating behaviour, there was a significant relationship between screen time and external eating with R^2^ = 0.1103, F = 12.357, *p* < 0.01. The interaction effect was not significant (X*W = 0.0021, *p* > 0.05). Apart from that, in this study, female adolescents (B_female_ = 0.1522, *p* < 0.01) had a positive and significant relationship between screen time and external eating compared to male adolescents. Interestingly, there was a significant unique variance in reported restricted eating R^2^ = 0.1292, F(1,302) = 14.78, *p* < 0.01. Nevertheless, the interaction term was not significant (X*W = 0.0011, *p* > 0.05). Female adolescents (B_female_ = 0.1911, *p* < 0.01) showed a higher relationship between screen time and restricted eating compared to male adolescents (B_male_ = 0.1328, *p* < 0.05). 

The moderation model used in Table 6 for significant variance among adolescents reported emotional eating R^2^ = 0.064, *p* < 0.001 with a combined effect of this magnitude (f^2^ = 0.018), considered a small-size effect. The interaction term (X*W) accounted for a significant 1.5% of the variance in the emotional eating variable F = 3.93, *p* < 0.05. The results indicate that for −1sd on the centre physical level representing low physical activity, the relationship between screen time and emotional eating was negative and not significant (*p* = 0.6168). Furthermore, at the medium physical activity or the centred moderator variable, the relationship was negative and not significant (*p* = 0.6168). However, at +1sd, representing high physical activity, the relationship was positive and significant (*p* = 0.003). Nevertheless, there was no significant interaction between the moderator variable (physical activity) and screen time for external and restricted eating behaviour. Meanwhile, for external eating behaviour, the low physical activity and medium physical activity as moderating variables indicated that there was a negative, but not significant (*p* > 0.05), relationship between screen time and eating behaviour. Furthermore, the high physical activity indicated there was a significant relationship between screen time and external eating behaviour (*p* = 0.031). In addition, the same pattern was also observed for restricted eating behaviour, whereby low and moderate physical activity showed no significant relationship between screen time and eating behaviour. Nonetheless, there was a positive and significant relationship between screen time and restricted eating for high levels of physical activity (*p* = 0.0001). 

## 4. Discussion

This study provides policy makers in the school sector with new information about how to better understand adolescents’ eating habits, screen usage and physical activity. The results of this study can also be utilised to enhance the role of parents and teachers in promoting physical exercise, healthy eating habits and efficient screen use, particularly in the wake of the COVID-19 pandemic. According to our findings, 99.5% of respondents had more than two hours screen time per day, including at the weekends and during weekdays. Moreover, the average amount of time spent in front of a screen per day throughout the weekday was 12 h, which is significantly more than the average of 9 h per day as reported in another study conducted in Malaysia during the COVID-19 pandemic lockdown [28,29]. These findings were comparable to those of other studies that found adolescents aged 11 to 12 years old spent more time watching television or watching movies compared with this study’s average of 129 min per day during the week. In the UK, a prior study has found that adolescents spent an average of 190 min per day watching television or movies [13]. 

A previous study has indicated the disadvantages of excessive screen time. For example, low self-esteem and an increased reliance on the internet, which can result in mental health issues, were concerns related to excessive screen time according to a study among adolescents in the Netherlands [30]. Additionally, it has been shown that excessive phone use among college students is associated with decreased levels of life satisfaction, poor academic results, and elevated anxiety. Excessive screen use was associated with behavioral, emotional, and psychological issues, according to a different meta-analysis study of six studies among adolescents [31,32]. Another study of adolescents found that spending more than an hour a day on screens was associated with poor psychological health, including lower self-control, difficulty making friends, emotional instability, frequent distraction, less curiosity about learning new things, and difficulty finishing tasks [33]. According to a 2019 study, deactivating social media accounts increased offline activities, such as spending time with family and friends, while decreasing insights into news based on social media as people rely more on news from radio, television, and newspapers [34].

In this study, the times spent on different types of screens were compared between genders, including playing console games on weekdays and weekends. This is consistent with earlier research conducted in Canada among children under the age of 18 years [35]. In that study, screen time included time spent using a computer, tablet, television and video game consoles. Based on the findings of this study, it can be concluded that eating behaviour has a substantial association with the amount of time spent playing video games on a console and using a desktop computer on a weekly basis. This is similar to a recent cross-sectional study that evaluated adolescents in Canada and discovered a substantial association between watching television and playing video games with emotional eating [16]. In addition, there was a strong correlation between the eating behaviour score at weekends and time spent watching television and using a laptop. This result was in line with a prospective cohort study in Spain that revealed a relationship between total screen time and the emotional, restricted and external eating behaviours of adolescents aged 8 to 10 years [36].

Past research has shown that cutting back on screen time, such as the amount of time spent on social media, can enhance offline activities such as exercise. Many advantages of exercise include a decreased chance of chronic diseases such as heart disease, hyperlipidemia, obesity, diabetes, and hypertension. Physical activity is advantageous in terms of how it affects hemorheological markers. These hemorheological variables can aid in facilitating smooth blood flow through the arteries. Moreover, specialised physical activity training can lower blood triglyceride levels while concurrently raising high-density lipoprotein cholesterol (HDL-C) and lowering low-density lipoprotein cholesterol (LDL-C). Additionally, the significance of preventing socially relevant illnesses such as arterial hypertension and atherosclerosis is intriguing [37,38]. Moreover, a certain type of fitness programme can normalize increased fibrinogen levels. A prior study found that a group of patients with cerebrovascular disease who exercised regularly for a month experienced considerably lower levels of fibrinogen [39]. According to a study among adults, those who are not physically active have higher plasma fibrinogen levels, a higher body mass index, and a higher total cholesterol level [40,41] In addition, increasing screen time could result in pathophysiological consequences on hemorheological markers from a lack of physical activity. This sedentary lifestyle may have an impact on oxidative stress, metabolic alterations in cells and tissues, changes in lung and respiratory functions, and adjustments to the control and tone of the blood vessels [42]. Hence, engaging in physical activity has several advantages, especially for adolescents, who may minimise their screen time or boost offline activity as well as enhance their nutritional intake.

A substantial association was also found in this study between the amount of physical activity a person engaged in throughout the weekday and the amount of time they spent using a tablet. In addition, over the weekend, there was a positive correlation between time spent watching television and using a laptop and a negative correlation between physical activity and time spent using a smartphone. A similar study conducted in Spain found that boys’ total screen time, including time spent watching television, using computers, playing video games and using portable mini consoles was significantly correlated with their physical activity, as measured by the Minnesota Leisure Time Physical Activity Questionnaire (MLTPAQ) [43]. In contrast to this finding, a study conducted among Polish children aged 18 years and below reported that lower daily physical activity times were observed among children with longer screen time [44]. Similarly, based on a study among children and adolescents in Iran, greater screen time, including watching television or using computer, was found to be associated with low engagement in physical activity [45].

There was a substantial association between emotional, restricted, and external eating behaviour and screen time in this study, which is another interesting finding for gender as a moderating factor. There was a substantial interaction impact between gender and screen time, but only for emotional eating. In this study, the association between screen time and emotional eating was found to be stronger in female adolescents than in male adolescents. This outcome was consistent with earlier studies, whereby there was a substantial association between emotional, restricted and external eating with screen time [35]. Emotional eating has a relationship with body image among female adolescents, based on a study in China [46]. In Malaysia, a previous study indicated there was a significant relationship between emotional eating and distorted body image, which may lead to unhealthy eating behaviour among adolescents [47]. One of the factors contributing to unhealthy eating in schools, according to a different study conducted among Malaysian schoolchildren, was the low cost of buying snacks and fried foods [48].

According to a study conducted in China among adolescents aged 11 to 17 years, there are significant differences in eating behaviour between genders as measured by the DEBQ, with a higher percentage of female adolescents reporting emotional, restrained, and external eating behaviour than male adolescents [49]. According to a different study, there was a significant interaction between gender and the frequency of eating fruit and vegetables. Male adolescents consume significantly more fruit and vegetables than female adolescents. In contrast to other ethnic groups, ethnic Asians and Latinos showed a significant association with emotional eating. Asian adolescents and Latino youth consume less fruit, vegetables, and salt, which is associated with higher levels of energy [18]. This was further corroborated by research among university students in Hong Kong, where it was discovered that female students had a much higher chance of negative emotional eating and higher odds ratios than male students [50]. In this study, there was significant mean difference in eating behaviour between genders, and this is related to other factors such as the location of the participants’ schools, frequency of eating out per week, frequency of snacks per week, reading habits regarding the information on food labels [51], and lifestyle behaviour [52]. Another study on emotional eating involved 12 nations including Australia, South Africa, China, India, Europe, and the United Kingdom. The findings showed a significant associations between emotional eating and various nations’ unhealthy dietary habits. Fast food, sugary drinks, sodas, fries, cakes, and ice cream are among the unhealthy food choices that were discovered to show similar eating patterns across all 12 countries [53].

In addition, this study has also revealed that there was a strong association between screen time and all dimensions of eating behaviour, utilising the degree of physical activity as the moderating effect. However, the interaction effect was notable for emotional eating. Additionally, the categories of active teenager had a stronger correlation between screen time and significant emotional eating. The results of this research were consistent with those of a prior study, in which a cluster of people practised healthy diets and active lifestyles. However, a study among adolescents that used the HELENA Dietary Assessment Tool and the IPAQ as instruments for physical activity and dietary assessment produced five clusters: unhealthy, sedentary, active, low-quality diet, inactive, high-quality diet and healthy [54]. Furthermore, a cross-sectional study conducted in Europe revealed that adolescents who were active consumed more nutritious foods [55]. This is also supported by previous study, in which less consumption of puffed products but greater intake of nuts, dried fruits and vegetables was described among physically active children in Eastern China [56]. This research was conducted in parallel with a study among youngsters in India, in which the authors discovered a strong relationship between screen time, physical activity and eating habits [57]. On the contrary, the findings of this study differed from a prior local study conducted among adolescents in Kuantan, Pahang, whereby the authors discovered that there was no significant correlation between physical activity and eating behaviour, as measured by an eating attitude test [58]. Another factor contributing to increased screen time and emotional eating during the data collection period was the pandemic, which caused adolescents to spend a lot of time viewing advertisements on the internet and television. In contrast to regular days, television advertisements for unhealthy foods and beverages predominated during school breaks [59]. The majority of teaching and learning activities during the COVID-19 pandemic were conducted online; therefore, adolescents were exposed to higher levels of screen time, particularly in the form of watching television and using the internet [60].

This study had a number of limitations. Respondents may not disclose their low levels of physical activity during the post-COVID 19 period because they have a poor understanding of the questions about physical activity. Second, some respondents might have underestimated their screen time for all household devices. Nonetheless, this study had a number of advantages, including a large sample size and data collection that occurred post-COVID-19. As a result, the information is important and essential for parents and policy makers, because earlier studies tended to be conducted before the pandemic. Future research should consider different food types and levels of stress, anxiety and depression. Future research should consider including biochemical parameters such as fibrinogen, pathophysiological effects, different food types and levels of stress, anxiety, and depression.

## 5. Conclusions

This study concludes that excessive screen time influences adolescents’ physical activity and eating behaviour. This varied by gender, with females seeing a higher association between screen time and eating behaviours, particularly emotional eating. This may increase the likelihood of becoming overweight or obese. As a result, an intervention was proposed to manage emotional, external and restricted eating behaviour among adolescents and encourage an active lifestyle along with the promotion of ‘effective screen time’ in gadget utilisation.

## Figures and Tables

**Table 1:** The profile of the respondents.

Variable	*n*	Percentage	Mean	SD
**Gender**				
Male	140	37.8		
Female	232	62.2		
**Age group**				
13–15	93	25		
16–17	279	75		
**Household income**				
<RM1000	92	26.1		
RM1001–RM3000	163	46.2		
RM3000–RM6000	66	18.7		
>RM6000	29	8.2		
**Race**				
Malay	300	85.0		
India	36	10.2		
Chinese	15	4.2		
Others	2	0.6		
**Numbers of devices**				
<3	234	62.9		
≥3	138	37.1		
Screen time				
<4 h	38	10.2		
≥4 h	334	90.8		
**Eating behaviour**				
Emotional			33.91	10.86
External			22.43	6.50
Restricted			21.62	7.45
**Physical activity**				
Low active	140	39.7		
Moderate	166	47.0		
Active	46	13.0		
**Total hours screen time** (weekday)			12.477	8.01
**Total hours screen time** (weekend)			13.877	8.35
**Screen time weekday >2 h**	372	99.4		
**Screen time weekend >2 h**	372	99.3		

**Table 2:** Mean difference for screen time among male and female adolescents during weekends and weekdays.

	Weekday				Weekend			
Gender	Male		Female		Male		Female	
Duration (Hours)	Mean	SD	Mean	SD	Mean	SD	Mean	SD
Telephone	7.39	3.87	6.96	3.85	7.37	3.78	6.96	3.86
Television	2.15	2.179	1.99	1.933	1.86	1.82	1.99	1.93
Tablet	0.43	1.379	0.46	1.106	0.34	1.324	0.48	1.36
Laptop	1.13	2.009	1.06	2.534	1.01	1.86	0.98	1.83
Desktop	0.44	1.294	0.44	1.499	0.44	1.49	0.30	0.81
Non-handheld console game	0.27 **	1.114	0.587 **	0.491	0.27 *	0.83	0.12 *	0.49
Handheld console game	0.26	0.93	0.15	0.724	0.16	0.89	0.15	0.72

* *p* < 0.05, ** *p* < 0.001.

**Table 3:** Mean difference for the eating behaviour domain and physical activity.

Gender	Male		Female	
	Mean	SD	Mean	SD
**Eating behaviour**	(*n* = 140)		(*n* = 232)	
**Emotional eating**	29.7000 **	9.95941	36.4526 **	10.61883
**External eating**	20.2500 **	6.51078	23.7500 **	6.15229
**Restricted eating**	18.7214 **	6.93346	23.3707 **	7.22134
**Physical activity**	2.5898 **	0.84395	2.2210 **	0.65624

** *p* < 0.001.

**Table 4:** Associations of screen time with eating habits and physical activity levels during weekdays and weekends among adolescents aged 13 to 17 years old in the Klang Valley, Malaysia.

Measurements	Eating Habits (Weekdays)	Eating Habits (Weekends)	Physical Activity (Weekdays)	Physical Activity (Weekends)
Duration (Hours)	Β(SE)	Β(SE)	Β(SE)	Β(SE)
Telephone	0.015 (0.008)	0.020 (0.011)	−0.018 (0.10)	−0.023 (0.009) *
Television	0.006 (0.014)	0.043 (0.020) *	0.010 (0.019)	0.031 (0.014) *
Tablet	−0.005 (0.023)	−0.053 (0.034)	0.112 (0.040) **	0.019 (0.037)
Laptop	0.027 (0.015)	0.058 (0.026) *	0.035 (0.020)	0.044 (0.019) *
Desktop	0.080 (0.037) *	0.130 (0.084)	−0.032 (0.053)	−0.052 (0.044)
Non-handheld game console	−0.090 (0.035) *	0.054 (0.108)	0.004 (0.043)	0.019 (0.055)
Handheld game console	0.041 (0.046)	−0.070 (0.067)	−0.113 (0.064)	−0.091(0.068)
Model summary	Adjusted R^2^ = 0.117 F = 2.825 *	Adjusted R^2^ = 0.117 F = 2.825 *	Adjusted R^2^ = 0.043 F = 2.872 **	Adjusted R^2^ = 0.036 F = 2.545 *

* *p* < 0.05; ** *p* < 0.01.

**Table 5:** Moderation model coefficients for screen time predicting eating behaviour, conditional on gender (*n* = 372), using standardised regression coefficients and 95% confidence interval.

Variable	B [LLCI, ULCI]	SE (HC3)	B [LLCI, ULCI]	SE (HC3)	B [LLCI, ULCI]	SE (HC3)
	DV = Eating Habits (Emotional)		DV = Eating Habits (External)		DV = Eating Habits (Restricted)	
Constant	22.3023 (18.312, 26.292)	2.02	16.045 (13.713, 18.377)	1.185	13.674 (11.037, 16.312)	1.340
Screen time	0.2792 (−0.772, 0.213)	0.2502	0.007 (−0.281, 0.295)	0.146	0.074 (−0.251, 0.399)	0.165
Gender	6.8095 (4.439, 9.180) **	1.2046	3.738 (2.353, 5.124) **	0.704	4.524 (2.958, 6.091) **	0.796
Screen time * Gender	0.3288 (0.051, 0.617) **	0.1466	0.072 (−0.096, 0.241)	0.085	0.058 (−0.1323, 0.249)	0.097

* *p* < 0.05, ** *p* < 0.01.

**Table 6:** Moderation model coefficients for screen time predicting eating behaviour, conditional on physical activity (*n* = 372), using standardised regression coefficients and a 95% confidence interval.

Variable	B [LLCI,ULCI]	SE (HC3)	B [LLCI,ULCI]	SE (HC3)	B [LLCI,ULCI]	SE (HC3)
	DV = Eating Habits (Emotional)		DV = Eating Habits (External)		DV = Eating Habits (Restricted)	
Constant	32.793 (31.481, 33.978)	0.634	21.789 (21.056, 22.522)	0.372	20.593 (19.779, 21.406)	0.413
Screen time	0.199 (−0.029, 0.427)	0.116	0.0878 (−0.047, 0.223)	0.069	0.161 (0.019, 0.303) *	0.072
Physical activity Level	2.589 (0.498, 4.678) *	1.061	1.724 (0.441, 3.007) **	0.652	2.062 (0.527, 3.597) **	0.779
Screen time * Physical activity level	0.257 (0.0018, 0.511) **	0.129	0.047 (−0.094, 0.189)	0.072	0.146 (−0.010, 0.302)	0.079

* *p* < 0.05, ** *p* < 0.001.

## Data Availability

The data are unavailable due to privacy.
